# Combination Effect of Deferoxamine and Arsenic Trioxide on Viability and Vitality of APL Like Cell Line

**DOI:** 10.4314/ejhs.v33i4.17

**Published:** 2023-07

**Authors:** Kazem Ghaffari, Ali Bayat, Ali Ghasemi

**Affiliations:** 1 Department of Basic and Laboratory of Sciences, Khomein University of Medical Sciences, Khomein, Iran; 2 Department of Biochemistry and Hematology, Semnan University of Medical Sciences, Semnan, Iran

**Keywords:** Acute Promyelocytic Leukemia, Arsenic trioxide, Deferoxamine

## Abstract

**Background:**

Arsenic trioxide is an activist agent in the treatment of acute promyelocytic leukemia (APL), which acts alone, but has an adverse effect on patients. Moreover, deferoxamine has antiproliferative activity and induces leukopenia. In order to enhance antileukemic effectiveness and to reduce the dosage of arsenic trioxide, the combination effect of it with deferoxamine (DFO) was evaluated on the APL cell line (NB4).

**Methods:**

In this experimental study, to investigate the cytotoxic effects of ATO/DFO in acute promyelocytic leukemia, the NB4 cell line (provided by Pasteur Institute of Iran) was treated with different doses and then at 24, 48, and 72 hrs intervals, the percentage of survival, cell count, metabolic activity and apoptosis induction were investigated respectively. Also, hTERT gene expression was analyzed by the RT-PCR method.

**Results:**

We found that DFO alone and in combination with ATO has cytotoxic and antiproliferative effects, and reduces viability and cell metabolic activity in the NB4 cell line in a dose and time-dependent manner. In addition, this combination causes an increase in apoptosis, up-regulation of Caspase-3, and down-regulation of hTERT genes in cells.

**Conclusion:**

Combined ATO/ DFO treatment cooperatively decreased the mRNA levels of the hTERT and increased the mRNA levels of Caspase-3 in a time-dependent manner compared to DFO alone.

## Introduction

Arsenic has been used as a therapeutic agent for more than 2400 years (1). In the 15th century, Hsin et al. discovered digitalis as a strong proponent of arsenic-based therapies. He argued, “Small doses of poisons are the best medicine, while too large doses of the best medicines are poisonous” (1). Antileukemic activity of arsenic was first reported in the late 1800s. A report from Boston City Hospital in 1878 described the effect of Fowler's solution, a solution containing arsenic, on the reduction of white blood cell count in two normal people and one patient with “leucocythemia” (2). Since then, As2O3 has been administered as a primary antileukemic agent until its replacement by radiation therapy. The hematologic use of arsenic, however, regained its popularity in the 1930s due to its efficacy in patients with chronic myelogenous leukemia (CML) (3).

Acute myeloid leukemia (AML) is a clonal hematopoietic disorder, characterized by uncontrolled self-renewal of hematopoietic stem cells, maturation arrest of myeloblast level, and blast cell infiltration to peripheral blood and bone marrow (4-10). In modern chemotherapy, arsenic trioxide after radiation is considered the most effective treatment for CML and other types of leukemia (11). The reports from China have shown that arsenic trioxide induces clinical and hematologic responses in patients with de novo conditions and relapsed acute promyelocytic leukemia (APL) (12). One report also indicated that arsenic trioxide monotherapy induced complete clinical responses in 9 of 10 patients with relapsed APL (13).

Other studies reported the use of arsenic compounds, such as ATO in the treatment of newly diagnosed and relapsed patients with APL, due to the ability of ATO to induce partial myeloid differentiation and caspase specific apoptosis (14,15). ATO induced complete remissions in 85% of relapsed cases and triggered apoptotic death in APL cells when used alone (16). Previous studies have reported that ATRA-ATO combined treatment regimens can eradicate leukemic progenitor cells and reduce the relapse rate to <10% even in high-risk APL patients (17, 18). Additionally, several studies have indicated that iron deprivation by chelation can contribute to cancer prevention and management. As a bacterial siderophore agent, DFO is an iron chelator with apoptotic effects on cells, in addition to anti-proliferative activity against several cancer cell lines (19-21). Accordingly, a combination of DFO and ATO can be a promising option for APL treatment. In this regard, the NB4 cell line was exposed to different doses of DFO alone and in combination with ATO. It was hypothesized that a combined treatment of DFO and ATO could increase ATO cytotoxicity. The results of the present study could be useful for the evaluation and selection of the best treatment strategies.

## Materials and Methods

**Cell culture and treatment**: Human APL cell line NB4 t (15; 17) was used in this study, and its PML/RARα expression was confirmed by RT-PCR. Cells were cultured by initial seeding of 4×106 cells/mL in RPMI 1640 medium (GIBCO-BRL) containing 10% heat-inactivated fetal bovine serum (GIBCO-BRL), 100 U/ml penicillin, and 100 mg/ml streptomycin (Bioidea) in a humidified incubator at 37 °C with 5% CO2. Then, a definite amount of stock ATO solution (0.5 mM in RPMI) (Sina Darou, Tehran, Iran) was added to a culture medium to reach the concentration of 0.5 μM and a solution of DFO (Sigma) with a concentration of 10 μM These solutions were prepared by dissolving the compounds in sterile PBS. They were then divided into aliquots and stored at −20 °C. NB4 cells were treated with proper amounts of DFO stock solution to attain different concentrations (50, 100, and 200 μM).

**Trypan blue exclusion test of cell viability**: The effects of DFO and/or ATO on cell growth were evaluated by seeding NB4 cells (1×10^5^ cells/mL) and their incubation with different concentrations of drugs for 24, 48, and 72 h. The cell suspension was then centrifuged and the cell pellet was resuspended in serum-free complete medium. One part of 0.4 % trypan blue (Invitrogen) was mixed with one part of cell suspension and incubated for 2 min at room temperature. The total number of unstained (viable) and stained (nonviable) cells was manually counted and determined. Finally, the percentage of viable cells was calculated by the following equation:


$$\text{ Viability }(\%)=\frac{\text{ viable cell count }}{\text{ total cell count }}\times 100.$$

**Micro culture Tetrazolium test**: The repressive effect of DFO, ATO, and their combination on the metabolic activity of NB4 cells were measured based on the reduction of (yellow color) 3-4, 5-dimethythiazol-2-yl)-2, 5- diphenyl tetrazolium bromide (MTT, Sigma) to Formosan (dark purple). Briefly, NB4 cells were seeded at 5000 cell/well in flat bottom 96-well culture and incubated with 50, 100, and 200 μM DFO and/or 0.5 μM ATO for 24, 48, and 72 h. After removing the medium, cells were further incubated with MTT solution (5 mg/ml in PBS) at 37 °C for 3 h. The untreated cells were defined as the control group. Supernatant medium was discarded after centrifugation, followed by adding DMSO to dissolve precipitated Formosan. Finally, the absorbance of the converted dye was measured with an ELISA reader at a wavelength of 570 nm.

**Measurement of caspase 3 enzymatic activity**: The enzymatic activity of caspase 3 was evaluated by a caspase 3 kit (Sigma, USA) to investigate the caspase dependence of ATO/DFO-induced cell death in the NB4 cell line. After the desired time and centrifugation at 600 g for 5 minutes to lyse the cell pellet, lysate buffer was added, followed by 10 minutes of centrifugation at 20000 g. The supernatant (5 μl) was incubated with 85 μl of assay buffer along with 10 μl of caspase 3 substrates for 2 h in an 83-well plate. The breakdown of the peptide by caspase 3 leads to the dye release, which can be detected by a spectrophotometer at a wavelength of 450 nm.

**Investigating hTERT gene expression in NB4 cell line**: RNA was isolated from NB4 cells after 24, 48, and 72 hours of using Trizol. The quantity of RNA samples was spectrophotometrically assessed by a NanodropND-1000 (Nanodrop Technologies, Wilmington, Delaware, USA). For cDNA synthesis, 1 μg of total RNA was reverse transcribed using the cDNA synthesis kit (Qiagen). Quantitative real-time RT-PCR was performed on a light cycler instrument (Roche Diagnostics, Mannheim, Germany) using the Eva Green Master Mix kit. Eva Green master mix (4 μl), 1-5 μl of cDNA samples, 0.5 μl of forward and reverse primers (10 pmol), and up to 20 μl of nuclease-free water (Qiagen, Hilden, Germany) were mixed in a capillary tube (Roche). PCR was performed as follows: initial activation for 30 s at 95 °C, followed by 45 cycles of denaturation at 95 °C for 5 s, and a combined annealing/extension step for 20 s at 60 °C. The sequences of primers are listed in Table 1. Glyceraldehyde 3-phosphate dehydrogenase (GAPDH) was used as a reference gene and the folded changes in the relative expression of each target mRNA were calculated based on the comparative Ct (2−ΔΔCt) method.

**Table 1:** Nucleotide sequences of the primers used for real-time RT-PCR

Gene	Forward primer (5′–3′)	Reverse primer (5′–3′)
Caspase 3	TTCAGAGGGGATCGTTGTAGAAGTC	CAAGCTTGTCGGCATACTGTTTCAG
hTERT	CGTCGTCGAGCTGCTCAGGTC	GGATGAAGCGGAGTCTGGACG
GAPDH	GAAGGTGAAGGTCGGAGTC	GAAGATGGTGATGGGATTTC

**Statistics**: Results were based on three independent experiments. Significant differences between the experimental variables were determined by a two-tailed Student's test (Microsoft Excel, Microsoft Corp). A probability level of P<0.05 was considered statistically significant.

**Ethics Approval**: This study was approved by the Ethics Committee of Tehran University of Medical Sciences (ethical committee code number: IR.TEHRANMU.REC.1392.02).

## Results

**Reduction of NB4cell count upon treatment with a combination of DFO and ATO**: The results showed that treatment with DFO remarkably declined the cell count of the NB4 cells in a dose- and time-dependent manner. The minimum number of cells was observed in the case of using 200 μM DFO in combination with 0.5 μM ATO for 72 h (Table 2).

**Table 2:** Cell count treated with DFO plus ATO

		cell count	
DFO and/or ATO	24 h	48 h	72 h
	
50 DFO	345000	168000	103000
100 DFO	329000	140000	91000
200 DFO	283000	111000	79000
0.5 ATO/50 DFO	267000	95000	66000
0.5 ATO/100 DFO	256000	80000	54000
0.5ATO/200DFO	248000	72000	38000
Control	464000	302000	200000

**Figure 1:**
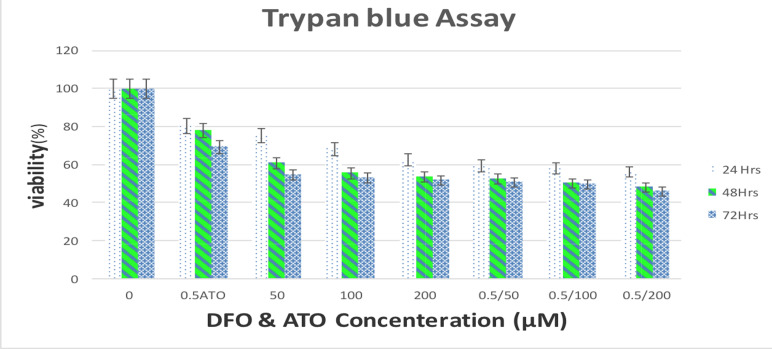
Effect of DFO and/or ATO on viability of NB4 cells. Cells were treated with designated concentrations of DFO and/or ATO for 24, 48, and 72 h; the inhibitory effects of DFO and/or ATO on NB4 cell viability were assessed with a trypan blue exclusion test

**Combination of DFO and ATO decreased cell viability in APL cells**: Viability assessment using trypan blue exclusion assay showed the effectiveness of doses of DFO in combination with ATO. Based on Figure 1, 24 hours of treatment with DFO at concentrations of 50, 100, and 200 μM decreased the viability of NB4 cells by 75.3, 68.3, and 62.6 %, respectively. This decline was 60.8, 55.5, and 53.7 % after 48 hours of treatment and 54.8, 53.2, and 51.7 % after 72 hours of treatment, respectively. In cells treated with DFO and ATO at concentrations of 50/0.5, 100/0.5, and 200/0.5 μM, these reductions were 59.4, 58.2, and 56.3 % after 24 h, 52.4, 50.2, and 48.1 % after 48 h, and 50.7, 49.7, and 46.1 % after 72 h, respectively (Figure 1).

**Figure 2:**
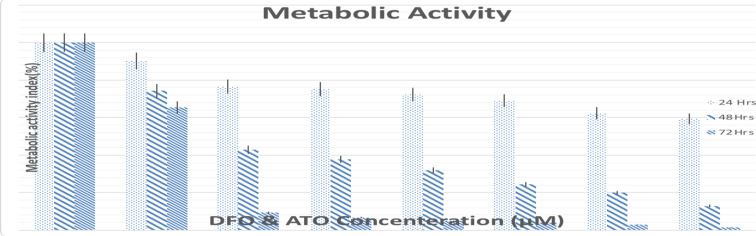
The effects of DFO and/or ATO on metabolic activity of NB4 cells. Metabolic activity was assessed with an MTT assay after 24, 48, and 72 h incubation with indicated concentrations of DFO and/or ATO

**DFO reduced metabolic activity of APL cells**: MTT assay was performed to investigate the metabolic activity of NB4 cells after 24, 48, and 72 hours of treatment with DFO and/or ATO. Treatment with all doses of DFO in combination with ATO was effective in a dose- and time-dependent manner. As shown in Figure 2, treatment with DFO at concentrations of 50, 100, and 200 μM decreased the metabolic activity by 76, 75.18, and 72.29 % after 24 h, 42.84, 37.59, and 31.95 % after 48 h, and 9.29, 6.73, and 4.87 % after 72 h, respectively. These reductions in the group treated with the combination of 0.5 μM ATO were 69.09, 62.3, and 59.28 after 24 h, 24.3, 19.89, and 12.9 % after 48 h, and 3.9, 2.79, and 1.42 % after 72 h, respectively (Figure 2).

**Figure 3:**
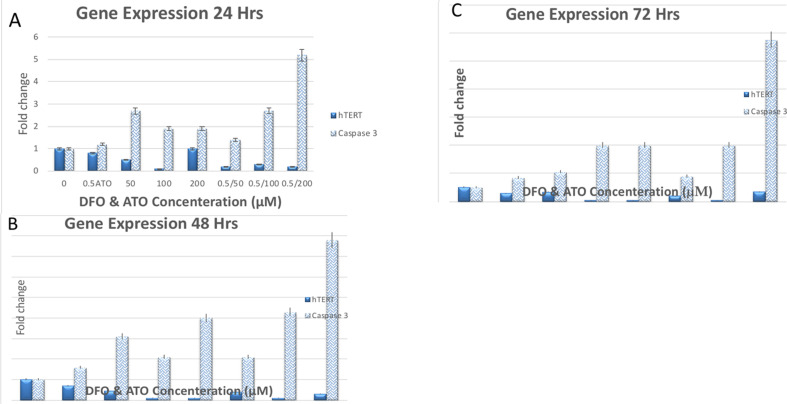
The effects of DFO and/or ATO on apoptosis-related genes. A) Gene expression after 24 h. B) Gene expression after 48 h. C) Gene expression after 72 h

**Effect of DFO and/or ATO on apoptosis-related genes**: The expression rate of telomerase-related gene of hTERT and Caspase-3at mRNA was evaluated by qRT-PCR after 24, 48, and 72 hours of incubation of NB4 cells that were treated with DFO (50, 100, and 200 μM) and/or ATO (0.5 μM). As shown in Figure 3, DFO suppressed the expression of hTERT and increased the expression of Caspase-3. Moreover, a combination of ATO and DFO cooperatively decremented the mRNA levels of hTERT while enhancing the mRNA levels of Caspase-3 more than DFO (Figure 3).

## Discussion

The results indicated that groups treated with combinations of DFO and ATO induced a remarkable reduction in the cell count of NB4 cells in a dose and time-dependent manner. Combinations of 200 μM DFO and 0.5 μM ATO for 72 hours led to the lowest cell count. Viability evaluations by trypan blue exclusion assay revealed that treatment with 200 μM DFO combined with 0.5 μM ATO for 72 h induced a drastic reduction in viability. An MTT assay was performed to investigate the metabolic activity of NB4 cells after 24, 48, and 72 hours of treatment with DFO and/or ATO. The combination of DFO (200 μM) with ATO (0.5 μM) for 72 h decreased the metabolic activity of the NB4 cells. Floros et al and Becton et al demonstrated the potent antitumor effects of DFO on neuroblastoma (NB) cell lines (22). Becton et al (23) showed the dose and time-dependent cytotoxicity of DFO on the NB cells, with maximal killing at an exposure dose of 50 μM DFO for 72 h. The complete proliferation inhibition was observed at concentrations above 20 μM.

In another study, Becton et al (24) indicated that DFO inhibits the proliferation of three human myeloid leukemia cell lines including HL-60, HEL, and U-937. Despite slight inhibition at DFO concentrations below 10 μM, maximal inhibition was recorded at DFO concentrations ≥20 μM. In addition, initial effects on proliferation were observed after 24 h of continuous exposure to DFO while the maximum effect occurred after 48 hours of treatment. Renton et al (25) demonstrated different effects of DFO on the proliferation of neural tumor cell lines depending on the drug concentration and exposure time. Scientific data have demonstrated that ATO-induced apoptosis is associated with up-regulation of the expression of the proenzymes of caspase 3 and activation of caspase 3 (26, 27). Moreover, Wang et al. reported that DFO induces apoptosis of HL-60 cells by activating caspase 3 (28).

The effects of DFO at concentrations of 10 and 160 μM were irreversible after 24 and 48 h, respectively, while 10 μM DFO became cytotoxic after 3 days. Furthermore, previous studies have described tumor-specific antiproliferative activity of DFO, with no impact on the human diploid cell line WI 38 (29, 30). In line with previous work, the result of the present study confirmed the antiproliferative effects of DFO (23-25). The findings of the present study demonstrated the benefits of the combination of DFO and ATO in combat against NB4 cells. Moreover, a combination of ATO and DFO had a higher impact on NB4 cells compared to their single use.

On the other hand, the expression rate of telomerase-related gene hTERT and Caspase-3 at mRNA levels showed that the combined ATO-DFO treatment cooperatively decreased the mRNA levels of hTERT and increased the mRNA levels of Caspase-3 in a time-dependent manner higher than DFO only. Based on the results of differential expression patterns in normal and tumor cells, telomerase could be a promising target for anticancer therapies (31). In addition, telomerase is an enzyme that maintains the length of chromosomal ends or telomeres, which otherwise would progressively shorten after each cell division. Some drugs have been designed to target various molecular components of telomerase, including hTERT (32). Chou et al (33) reported a decline in the hTERT mRNA levels of NB4 cells after 8 days of treatment with ATO (0.75 μM). Previous studies have reported that low doses of ATO (<0.5 μM) can induce differentiation in NB4 APL cells similar to ATRA (34), while apoptosis induction can be detected at higher concentrations (>1 μM) (35). Some of the apoptotic activities of ATO are related to the elevation of the intracellular ROS, reduction of mitochondrial transmembrane potential, and down-regulation of Bcl-2, leading to cytochrome c release and finally caspase-3 activation (36, 37). On the other hand, Wang et al (38) reported that DFO induces apoptosis of HL-60 cells based on activating caspase-3. This condition increases the apoptotic rate in a dose and time-dependent manner.

In conclusion, a combination of ATO and DFO could offer a promising therapeutic approach for treatment of APL patients. ATO and DFO seem to have synergistic mechanisms. Moreover, DFO, in combination with a low dose and non-apoptotic ATO, enhances the cytotoxicity of ATO, presenting an effective treatment for APL. Large clinical trial studies are, however, required to confirm the mentioned results.
